# Population Characteristics and Needs of Informal Caregivers Associated With the Risk of Perceiving a High Burden: A Cross-Sectional Study

**DOI:** 10.1177/0046958018775570

**Published:** 2018-05-29

**Authors:** Lotte Prevo, KlaasJan Hajema, Evelyne Linssen, Stef Kremers, Rik Crutzen, Francine Schneider

**Affiliations:** 1 Maastricht University, The Netherlands; 2 Public Health Service Southern Limburg, The Netherlands

**Keywords:** informal caregivers, perceived burden, cross-sectional study, population characteristics, needs

## Abstract

This study explored the population characteristics and needs of informal caregivers reporting a low or high burden. A cross-sectional study was conducted in the Netherlands to explore the associations between the characteristics and needs of informal caregivers and the burden they perceive and to assess the variance in perceived burdens that is explained by these variables. Three thousand sixty-seven adult informal caregivers and 1936 senior informal caregivers participated, almost 15% of whom perceived a high burden. Particularly caregivers in the 40 to 54 age group perceived a high burden, while caregivers with an intermediate educational level reported a low burden. Higher burden was also reported by caregivers who spent more time on the care provision tasks, had a high level of depressive symptoms, or reported loneliness. The explored variables seem to be important to explain caregiver burden. Longitudinal research is warranted to establish the causal directions of these associations.

**What do we already know about this topic?** Although there is considerable interest in possibilities to enhance informal care, the risk of a negative health impact among informal caregivers should be taken into account especially when the intensity of the care increases.

**How does your research contribute to the field?** This study provides a broad overview of characteristics and needs of informal caregivers that supports researchers in selecting risk factors for perceiving a high burden to eventually prevent overburdening.

**What are your research’s implications toward theory, practice, or policy?** To relieve the perceived burden of informal caregivers, a focus should be set on sharing care tasks, where a better cooperation with and support from the concerning partners in the municipalities is a necessity.

## Introduction

The problem of overburdening among informal caregivers has been increasing in developed countries, such as the Netherlands, where the number of caregivers reporting to perceive a high burden has been increased by 50% in the period from 2001 to 2008.^1^ In the Netherlands, recent changes introduced by the Dutch government, such as the objective of developing a “participatory society”^2^ and allocating more of the coordinating tasks to municipal authorities under the Social Support Act,^3^ have led to a greater focus on community development. Governments of other developed countries are also encouraging citizens’ initiatives to support each other.^4^ One of these initiatives that is becoming more popular these days concerns a greater focus on the possibilities of informal caregivers to take over formal care provision tasks.^5^ Informal caregivers are unpaid nonprofessionals who support chronically ill, disabled, and other people in need in their immediate environment, such as family members, friends, or neighbors, over a lengthy period of time.^6^ Caregivers often provide the support, because of their personal connection to the person in need.^7^ The support they provide can be physical, social, and/or emotional support, which is based on the care recipients’ needs and the abilities, capacity and willingness of the caregiver to provide the different types of support.^8,9^

In less intensive stages of caregiving, informal care is often perceived to be a source of positive influence on the lives of caregivers.^10^ The Dutch Expertise Centre for Informal Care^11^ found that informal caregivers often experience a sense of meaningfulness in life and feel more positive about themselves. Caregivers who receive some form of appreciation for their activities from the care recipient also mentioned positive influences of their care provision activities.^12^ However, as the intensity of the care increases, more caregivers report to perceive high burdens. When the caregiver burden, defined as a multidimensional response, eg, physical or mental, to the negative appraisal and perceived stress resulting from taking care of an ill individual in their immediate environment, becomes too intense, informal caregivers will be unable to provide the care.^13^ The perception of a high burden, when a caregiver is subjected to an excessive level of burden, has a negative influence on their physical, psychological, emotional, and functional state of health.^10^ A significantly lower quality of life was found among informal caregivers,^14^ which is also reflected by other indicators (eg, mental health problems such as stress, tension, anxiety and depression), and physical health problems (such as back injury, sleep disruption and hypertension).^7,10,15^

According to the Dutch National Institute for Public Health and the Environment, reporting a high burden is a predictor of an impending decline of the health status of caregivers.^16^ Based on Pearlin’s stress process model and various studies researching the topic of informal care, different characteristics can be identified that can explain a caregiver’s burden. First, background factors were seen as important, eg, the context of the caregiving situation and personal characteristics. A higher perceived burden was found to be associated with contextual factors such as collectivistic cultures that value the group as a whole,^7,17^ the nonexistence of a social care system,^18^ coresidence with the care recipient,^19^ and a small social network with low levels of social support.^20^ At a personal level, a high perceived burden was associated with a higher age,^19^ female gender,^20-22^ a lower socioeconomic status,^7^ a lower educational level,^23^ and poorer physical or mental health status of the caregiver.^20^ In addition, stressors, such as relationship factors,^7,10^ role strains,^24,25^ and psychological strains,^20^ were found to contribute to the burden perceived by informal caregivers. Mediating factors such as well-developed coping resources might reduce the influence of stressors.^19,26^ The specific combination of a caregiver’s background, stressors, and mediators determines the physical and mental health outcomes and the burden they perceive as shown in Pearlin’s stress process model.^20^

Although studies have focused on identifying important characteristics, a comprehensive overview aimed at exploring the involvement of all possible characteristics contributing to the risk of perceiving a high burden is currently lacking. In view of the increased recognition of the importance of supporting caregivers, it is necessary to be aware of potential risk factors. This study supports researchers in selecting these risk factors for perceiving a high burden. The considerable number of interventions that are available to support informal caregivers, such as training and education programs, approaches to care planning, support groups, individual counseling and mindfulness,^7,27-30^ specifically enhancing coping styles, can be more targeted at the risk factors among caregivers, which should eventually prevent overburdening.

The aim of this study was to explore the characteristics and needs of adult and senior informal caregivers reporting a low or high burden, as well as the contributions made by these characteristics to perceiving a high burden. These insights are essential for the development of future evidence-based interventions to reduce the burden perceived by informal caregivers.

## Methods

### Study Design, Participants, Recruitment, and Data Collection Procedure

This quantitative study had a cross-sectional design. In this study, secondary data analyses were performed on the gathered data from the Limburg Health Monitor 2012. The monitor is a nationwide survey that is repeated every 4 years, where information is collected from a representative sample of adult and senior Dutch citizens. It provides the Dutch government with information about the overall health status of Dutch citizens, including physical, mental, and social health domains, and based on this information, health policy targets are set.^31^ The Health Monitor is performed among two samples: adults (17-64 years) and seniors (65+). From all adults and seniors who participated, only informal caregivers were selected and included in this study. To answer the current research aims, data from both samples, adults and seniors, were used. Selected citizens received the Health Monitor questionnaire and an information letter at home by the end of September 2012.^32^ Participants who were providing informal care were included in the present study.

### Data, Instruments, and Measures

The question, “Are you currently providing informal care?” (0 = yes; 1 = no) was used to include all informal caregivers. The main outcome was the burden perceived by the respondent, as assessed by the question, “Do you currently feel burdened?” rated on a 5-point scale from perceiving no burden to being overburdened, which was recoded into low burden (0 = no, little, or some burden perceived) and high burden (1 = relatively severe or very severe perceived burden or being overburdened). Other assessed variables are described below, most of them based on validated indicator scales.

The background variables were age (1 = 17-24; 2 = 25-39; 3 = 40-54; 4 = 55-64; 5 = 65-74; 6 = 75-79; 7 = 80+), gender (1 = male; 2 = female), education (1 = low, ie, “primary, basic vocational, lower general school, or no education”; 2 = intermediate I, ie, “higher secondary education, preparatory academic education, or medium vocational school”; 3 = intermediate II, ie, “higher vocational school”; 4 = high, ie, “university level”), annual income (1 = maximum of 15 200 euros; 2 = 15 201-19 400 euros; 3 = 19 401-24 200 euros; 4 = 24 201-31 100 euros; 5 = minimum of 31 101 euros), marital status (1 = married/registered partnership; 2 = unmarried; 3 = divorced; 4 = widow/widower), and social contacts with family, friends, or neighbors (1 = at least once a week; 2 = less than once a week), social network based on Wenger’s classification^33^ (1 = locally integrated; 2 = family dependent; 3 = local self-contained; 4 = wider community focused; 5 = private restricted).

Three relationship factors for informal care provision, explaining the connection between informal caregiver and care recipient, were included. The first was the type of support provided, “Which activities do you carry out?” with answering options as follows: support for housekeeping, preparing meals, support for personal care, support for medical care, company, consolation and distraction, accompaniment and transport, administrative support, and other (0 = not providing the activity; 1 = providing the activity). The second factor was the recipient of the care provided, “To whom are you providing informal care?” with answering options as follows: partner, child, parent (or in-law), other family members, and neighbors/friends (0 = not providing care to the recipient; 1 = providing care to the recipient). The third was the duration of the care, “For how long have you been providing informal care?” with options shorter or longer than 3 months (1 = less than 3 months; 2 = more than 3 months), and “How many hours a week do you spend on providing care?” where caregivers had to fill in the mean number of hours, which was recoded into 3 categories (1 = 1-5 hours a week; 2 = 6-15 hours a week; 3 = >16 hours a week).

The physical and mental health status of the informal caregivers were assessed using 4 indicator scales. Quality of life was assessed by the question, “How good is your health?” (1 = good or very good perceived health; 2 = poor or very poor to moderate perceived health). Chronic conditions were assessed by presenting respondents with a list of conditions from which they could select those they suffered from (0 = no chronic conditions; 1 = one; 2 = two; 3 = three; 4 = four or more). Fear and depression were assessed with the Kessler Psychological Distress Scale, which includes 10 questions to be answered on a 5-point scale from always to never, indicating depressive symptoms, eg, feeling sad, restless, worthless, or tired, recoded into 3 categories (1 = no low symptoms; 2 = moderate symptoms; 3 = high symptoms).^34^ Loneliness was assessed with 11 questions to be answered on a 3-point scale (yes–more or less–no) for perceiving loneliness, eg, lacking a good friend, having a small social network, feeling of emptiness, and feeling abandoned, recoded into 3 categories (1 = no low; 2 = moderate; 3 = severe).^35^

Assessment of caregivers’ social roles focused on work situation, ie, having a paid job, by asking, “Which situation applies to you?” with answering options of having a paid job for <12 hours, 12 to 20 hours, 20 to 32 hours, >32 hours a week, being retired, unemployed, incapacitated, on social benefit, being homemaker, and being a student, (0 = unemployed, people without a paid job; 1 = employed, people with a paid job). Financial difficulties were also assessed: “Did you experienced financial difficulties in the last 12 months?” with the answers of no difficulties at all, no difficulties but I need to watch my expenditures, yes some difficulties, and yes many difficulties (0 = no difficulties; 1 = some and many difficulties). Family life was assessed by checking whether the caregivers’ households included children living at home, using the question, “With whom are you living together?” with answering options such as partner, children younger than 18 years, children aged 18 years and above, parent(s), other adult(s), and living alone (0 = no children at home; 1 = children at home).

The assessed coping indicators concerned mastery and self-management. Mastery was assessed using 7 questions on a 5-point scale from totally agree to totally disagree indicating the feeling of self-control, eg, ability to solve problems, control over things that happen in life, ability to control the future, recoded into low and high sense of mastery (1 = low; 2 = high).^36^ Self-management was assessed using 6 questions on a 6-point scale from never to very often, regarding self-management activities of the informal caregivers, such as taking the initiative to enter into or maintain contact with other people and actively engaging in leisure activities, recoded into 3 categories (1 = low; 2 = moderate; 3 = high).^37^

Finally, the needs of the caregivers were assessed by asking about 5 practical and emotional needs, eg, for information and advice regarding the execution of their caregiver role, replacement to take over care provision tasks, emotional support, relaxing activities, and advocacy regarding the representation and fulfillment of the interests of caregivers. The question was, “Apart from the support that you might already have, do you need another kind of support to help you with your caregiving tasks?” (0 = no need; 1 = need).

### Data Processing and Analysis

Bivariate analyses were performed, using Pearson’s chi-square, to explore the associations between perceiving a high burden and each of the variables described above. Subsequently, a logistic regression analysis was performed. The specific sampling, used to optimize the representativeness of the sample, needed to be taken into account during the analysis, which was done by carrying out the logistic regression analysis using a complex sampling procedure. Perceived burden among caregivers was included as the dependent variable and the other variables were selected as independent variables. Odds ratios (ORs) are reported as effect sizes and classified according to the recommendations by Rosenthal,^38^ where OR < 1.5 indicates a weak association, 1.5 to 2.5 a moderate association, 2.5 to 4.0 a strong association, and >4.0 a very strong association. The analysis contained 6 different categories of variables, the background variables, relationship indicators, roles of caregivers, physical and mental health status, and coping variables. All the analyses were performed with SPSS IBM statistics version 21.

## Results

### Quantitative Analysis

#### Description of the sample

From all participants, adults and seniors, who filled in the questionnaire of the Limburg Health Monitor, 3067 (12.8%) adult and 1936 (13.6%) senior participants provided informal care and could be included in this study (Table 1). The majority of the adults were women over the age of 40. Most participants were married or were living together with a partner, had an intermediate or high educational level, a paid job, and an annual income over 24 201 euros. Most of the senior participants were aged between 65 and 74, and a small majority of the participants were women. Most were married or were living together with a partner, had an intermediate educational level, an annual income below 24 200 euros, and were not in work. About two-thirds of the senior caregivers were retired. Almost 15% of all caregivers perceived a high burden due to their caregiving tasks.

**Table 1.** Description of Informal Caregivers Participating in the Study.

	Adult caregivers (n = 3067, 12.8%)	Senior caregivers (n = 1936, 13.6%)
Age, %		
- 17-24	177 (5.8)	—
- 25-39	322 (10.5)	—
- 40-54	1431 (46.7)	—
- 55-64	1137 (37.1)	—
- 65-74	—	1116 (57.6)
- 75-79	—	482 (24.9)
- 80+	—	338 (17.5)
Gender, n (%)		
- Male	935 (30.5)	941 (48.6)
- Female	2132 (69.5)	995 (51.4)
Education, n (%)		
- Low	87 (2.8)	263 (13.6)
- Intermediate I	882 (28.8)	892 (46.1)
- Intermediate II	1178 (38.4)	397 (20.5)
- High	920 (30.0)	384 (19.8)
Household income, n (%)		
- Maximum of €15 200	314 (10.2)	160 (8.3)
- €15 201-€19 400	452 (14.8)	503 (26.0)
- €19 401-€24 200	621 (20.2)	528 (27.3)
- €24 201-€31 100	846 (27.6)	424 (21.9)
- Minimum of €31 101	834 (27.2)	321 (16.6)
Employment status, n (%)		
- Not currently employed	902 (29.4)	1839 (95.0)
- Employed	2165 (70.6)	97 (5.0)
Marital status, n (%)		
- Married/living with partner	2441 (77.5)	1573 (81.3)
- Never been married	370 (11.8)	61 (3.2)
- Widowed	71 (2.3)	65 (3.4)
- Divorced	266 (8.4)	237 (12.2)
Burden perceived, n (%)		
- Low	2632 (85.8)	1650 (85.2)
- High	435 (14.2)	286 (14.8)

#### Associations between the burden perceived and the other characteristics

As regards the background variables, informal caregivers who had a lower educational level, a lower household income, a smaller social network centered on family, and privacy restricted, and caregivers who were widowed or divorced, perceived a significantly higher burden. Female gender was associated with perceiving a high burden among the adult caregivers (Table 2).

**Table 2.** Associations Between the Burden Perceived by Informal Caregivers and Their Background Variables.

Background variables	Adults (n = 3067)	Chi-square	Senior (n = 1936)	Chi-square
	Caregivers with a high burden		Caregivers with a high burden%	
Age, %				
17-24	5.4	853.30*		
25-39	12.8			
40-54	18.1			
55-64	11.5			
65-74			12.3	64.76*
75-79			17.1	
80+			14.5	
Gender, %				
Male	11.8	273.62*	13.8	0.95
Female	16.1		13.3	
Education, %				
Low	24.6	236.30*	21.6	196.90*
Intermediate I	14.0		13.7	
Intermediate II	13.6		11.5	
High	15.4		10.6	
Household income, %				
Maximum of €15 200	21.4	558.46*	16.2	99.57*
€15 201-€19 400	15.1		16.6	
€19 401-€24 200	13.8		12.8	
€24 201-€31 100	15.0		11.9	
Minimum of €31 101	11.3		10.2	
Marital status, %				
Married/living with partner	13.5	567.54*	14.7	107.59*
Never been married	13.7		10.7	
Widowed	22.3		7.5	
Divorced	23.5		11.8	
Social contacts, %				
Contacts with family: at least once a week	14.3	22.15*		
Contacts with family: less than once a week	16.3			
Contacts with friends: at least once a week	13.3	277.55*		
Contacts with friends: less than once a week	17.9			
Contacts with neighbors: at least once a week	14.2	13.67*		
Contacts with neighbors: less than once a week	15.1			
Network type, %				
Locally integrated			11.0	156.33*
Family-dependent			18.2	
Local self-contained			12.9	
Focused on wider community			10.2	
Private restricted			16.3	

* *P* < .05.

As regards the relationship factors, there was a positive association between perceiving a high burden and all caregiving activities, especially when more activities were provided. Providing care to a partner or children was associated with significantly higher burden, while care for parents (or in-laws), other family members, neighbors, and friends was associated with a low burden. The duration of the care provision also turned out to be a significant factor, as a larger number of hours per week and a longer duration were associated with a higher burden.

Role variables of the informal caregivers showed positive associations with a high burden being perceived by adult caregivers with children living at home, but this association was not found among the senior caregivers. Associations with social roles revealed a lower burden among employed caregivers. Finally, a high burden was found among adult and senior caregivers who had financial difficulties.

As regards the physical health of adult and senior informal caregivers, a lower perceived health status, a higher number of chronic conditions, and the presence of a long-lasting disease in the last 12 months were associated with a high burden. Among all caregivers, experiencing depressive symptoms and loneliness was associated with perceiving a high burden.

Caregivers who expressed a need for support, both practical and emotional, perceived a high burden. The strongest association was found when caregivers expressed a need for replacement and advocacy.

Finally, a low sense of mastery (all caregivers) and low self-management (senior caregivers) were associated with a high perceived burden.

An overview of the bivariate analysis of the characteristics and needs is provided in Table 3.

**Table 3.** Associations Between the Burden Perceived by Informal Caregivers and Their Characteristics and Needs.

	Adults (n = 3067)	Chi-square	Senior (n = 1936)	Chi-square
	Caregivers with a high burden		Caregivers with a high burden	
Relationship variables between caregiver and care recipient				
Activities				
Number of activities, %				
1	10.8	9173.69*	7.5	2241.10*
2	6.6		3.9	
3	9.3		6.8	
4	16.5		20.8	
5	32.6		23.0	
6	50.6		31.1	
7	48.1		51.2	
Practical support, %				
Yes	14.7	9.66*	15.1	134.28*
No	13.6		7.8	
Personal and medical care support, %				
Yes	29.9	3894.87*	25.9	997.22*
No	10.6		8.9	
Emotional support, %				
Yes	15.5	146.13*	16.6	238.55*
No	12.2		8.8	
Recipient				
Partner, %				
Yes	27.4	1306.50*	21.0	748.68*
No	13.0		7.9	
Child, %				
Yes	32.9	2576.80*	15.5	10.62*
No	12.4		13.2	
Parents (-in-law), %				
Yes	13.5	122.48*	11.1	14.77*
No	16.3		13.8	
Other family members, %				
Yes	9.8	294.14*	7.3	172.97*
No	15.5		15.1	
Neighbors, friends, %				
Yes	8.1	373.41*	3.2	504.97*
No	15.5		16.3	
Hours per week, %				
1-5 hours	6.6	7346.92*	4.2	1935.21*
6-15 hours	19.0		11.6	
>16 hours	36.5		19.1	
Duration, %				
<3 months	12.1	21.47*	6.5	39.26*
>3 months	14.7		13.7	
Roles of informal caregivers				
Living situation, %				
Families with children living at home				
Yes	16.8	297.16*	13.4	0.01
No	12.6		13.5	
Employment status, %				
Not currently employed	17.6	256.52*	13.6	2.59
Employed	13.3		11.8	
Financial difficulties, %				
Yes	22.3	1231.26*	20.0	147.61*
No	12.1		12.1	
Physical health of the informal caregiver				
Perceived health status, %				
(Very) poor	11.6	1888.41*	7.7	879.52*
(Very) bad	24.2		22.0	
Number of chronic conditions, %				
None	9.1	1626.13*	5.3	531.69*
1	13.1		9.5	
2	18.1		14.2	
3	19.3		17.8	
4	24.6		21.8	
Mental health				
Experiencing depressive symptoms, %				
None or low symptoms	8.5	5418.80*	5.5	2134.79*
Moderate symptoms	19.0		21.0	
High symptoms	44.5		48.5	
Loneliness, %				
None	9.2	4203.01*	8.2	1085.37*
Moderate	19.8		16.4	
(Very) severe	35.6		34.8	
Needs of informal caregivers				
No need for support, %				
Yes	9.3	10,584.34*	6.7	3902.44*
No	46.6		46.8	
Need for information and advice, %				
Yes	43.6	4413.62*	40.0	1142.34*
No	12.1		10.9	
Need for replacement, %				
Yes	64.9	5027.75*	60.6	2394.21*
No	12.9		10.5	
Need for emotional support, %				
Yes	50.7	3147.69*	48.6	887.20*
No	13.1		11.9	
Need for relaxing activities, %				
Yes	50.4	2602.38*	54.9	1145.88*
No	13.3		11.8	
Need for advocacy, %				
Yes	60.0	2383.41*	58.3	1073.06*
No	13.6		12.0	
Coping variables				
Sense of mastery, %				
Low	33.0	2033.79*	35.3	1403.70*
High	12.9		9.7	
Sense of self-management, %				
Low			26.1	619.28*
Moderate			11.7	
High			10.0	

* *P* < .05.

#### Logistic regression analysis

For the adult informal caregivers, the final model accounted for 30.2% of the explained variance in perceiving low or high burden. With regard to the background variables, being a female caregiver was moderately associated with perceiving a high burden, while being 50 to 54 years of age and being widowed were strongly associated with perceiving a high burden. Having an intermediate I and II educational level seemed to be associated with a low burden. The relationship variables of providing personal and medical care were moderately associated with a high burden, while more hours of care provision per week was strongly associated with perceiving a high burden. Although role factors turned out to be nonsignificant, experiencing many depressive symptoms was strongly associated with a high burden. A moderate/severe level of loneliness was moderately associated with a high burden among caregivers, while perceived health status and mastery were not significantly associated with perceiving a high burden.

Among the senior informal caregivers, the model explained 36.1% of the variance. Of the background variables assessed, only an intermediate II educational level was moderately associated with perceiving a low burden. The relationship variables of providing emotional support and personal/medical care support were moderately associated with perceiving a high burden. When more time was spent on providing care, this was strongly associated with a high burden. Although the role factors and perceived health status were not significantly associated with a high burden, a moderate/high number of depressive symptoms (strongly associated) and a severe loneliness level (moderately associated) were. Finally, a high sense of mastery was found to have a strong favorable association with the burden being perceived. An overview can be found in Table 4.

**Table 4.** Logistic Regression Analysis of Characteristics of Informal Caregivers With Regard to Perceiving a High Burden.

Independent variables	High burden among adults (n = 3067)		High burden among senior caregivers (n = 1936)	
	Model pseudo *R*^2^ = 0.302		Model pseudo *R*^2^ = 0.361	
	OR	95% CI	OR	95% CI
Background variables				
Age				
25-39 vs 17-24	1.33	(0.57-3.11)		
40-54 vs 17-24	3.03	(1.31-7.01)***		
55-64 vs 17-24	1.90	(0.75-4.81)		
75-79 vs 65-74			1.08	(0.70-1.69)
80+ vs 65-74			0.69	(0.15-3.08)
Gender				
Female vs male	1.50	(1.02-2.22)**	0.90	(0.58-1.40)
Education				
Intermediate II vs high	0.63	(0.42-0.97)**	0.49	(0.26-0.93)**
Intermediate I vs high	0.49	(0.31-0.77)**	0.55	(0.30-1.02)
Low vs high	0.59	(0.14-2.46)	0.65	(0.33-1.29)
Financial difficulties				
Yes vs no	1.10	(0.77-1.56)	1.04	(0.60-1.79)
Marital status				
Never married vs married	1.41	(0.72-2.75)	1.20	(0.33-4.43)
Divorced vs married	1.10	(0.65-1.87)	1.14	(0.35-3.66)
Widowed vs married	2.57	(1.10-6.04)***	0.69	(0.26-1.84)
Contacts with family				
<Once a week vs ≥once a week	0.97	(0.51-1.82)		
Contacts with friends				
<Once a week vs ≥once a week	1.06	(0.75-1.51)		
Contacts with neighbors				
<Once a week vs ≥once a week	0.76	(0.53-1.09)		
Network type				
Family-dependent vs locally integrated			1.27	(0.75-2.15)
Local self-contained vs locally integrated			0.84	(0.47-1.50)
Focused on wider community vs locally integrated			0.67	(0.21-2.12)
Private restricted vs locally integrated			0.73	(0.29-1.86)
Relationship variables				
Practical support				
Yes vs no	1.23	(0.83-1.82)	1.10	(0.52-2.32)
Personal and medical care support				
Yes vs no	2.13	(1.43-3.18)**	1.88	(1.22-2.90)**
Emotional support				
Yes vs no	1.01	(0.56-1.83)	1.73	(1.13-2.76)**
Recipient 1				
Partner: yes vs no	1.28	(0.73-2.25)	1.29	(0.54-3.08)
Recipient 2				
Child: yes vs no	1.45	(0.88-2.37)	1.57	(0.66-3.77)
Recipient 3				
Parents (-in-law): yes vs no	1.28	(0.83-1.99)	2.72	(0.96-7.72)
Recipient 4				
Other family members: yes vs no	0.86	(0.51-1.44)	1.72	(0.72-4.11)
Recipient 5				
Neighbors, friends: yes vs no	0.54	(0.28-1.02)	0.53	(0.25-1.11)
Hours per week				
6-15 hours vs 1-5 hours	2.60	(1.75-3.86)***	2.02	(1.04-3.93)**
>16 hours vs 1-5 hours	5.41	(3.29-8.89)****	6.27	(3.13-12.55)****
Duration				
>3 months vs <3 months	0.98	(0.49-1.95)	2.16	(0.72-6.50)
Roles, physical health, mental health of the caregiver				
Families with children at home				
Yes vs no	1.30	(0.88-1.91)	0.67	(0.24-1.86)
Employment status				
Employed vs not currently employed	1.01	(0.67-1.52)	1.68	(0.74-3.84)
Perceived health status				
(very) bad vs (very) good	1.41	(0.98-2.02)	1.34	(0.80-2.23)
Experience of depressive symptoms				
Moderate vs none or low	1.45	(0.93-2.26)	3.24	(1.94-5.41)***
High vs none or low	3.15	(1.66-5.97)***	5.79	(2.40-14.00)****
Loneliness				
Moderate vs none	1.76	(1.11-2.80)**	1.38	(0.85-2.22)
(very) severe vs none	2.21	(1.23-3.96)**	2.28	(1.23-4.21)**
Coping variables				
Sense of mastery				
Yes vs no	0.73	(0.25-2.15)	0.36	(0.15-0.86)***
Sense of self-management				
Moderate vs low			0.95	(0.41-1.28)
High vs low			1.18	(0.62-2.23)

*Note.* OR = odds ratio; CI = confidence interval.

* Small (OR < 1.5). **Moderate (OR 1.5 ≤ 2.5). ***Strong (OR 2.5 ≤ 4.0). ****Very strong (OR > 4.0).

## Discussion

This study explored the characteristics and needs of informal caregivers regarding their perceived burden. This section summarizes the main findings based on the variables included in the statistical analysis, that is the personal, contextual, relational and coping variables, and the needs of informal caregivers, and compares them with the findings of previous studies.

As regards the personal factors, a moderate association with perceiving a high burden was found for female gender, while strong associations were found for caregivers aged 40 to 54 years and those who were widowed. A moderate association with perceiving a low burden and intermediate levels of education was determined. This is in line with what was reported by others.^22,39,40^ Female caregivers from the “sandwich generation,” ie, those who need to take care of their own children while supporting their parents,^40^ were found to perceive a high burden. Caregivers with an intermediate educational level perceived a low burden. They are likely to be better able to handle the caregiving tasks, while feeling that their intellectual capacities are left unused.^41^ Widowed caregivers perceived a high burden, probably because of their lower level of coping resources to address the current caregiving tasks. They might be unable to share their tasks and thoughts or were still experiencing grief and bereavement because of a spouse who had died.^39,42^

Among the senior informal caregivers (aged 65 and above), only a moderate association was found between an intermediate educational level and perceiving a low burden, as was also reported.^41^ A smaller social network mainly dependent on family members, eg, close family ties with few neighbors and peripheral friends, or focused on privacy, eg, absence of relatives and friends nearby and low levels of community involvement,^43^ and feelings of loneliness were associated with perceiving a high burden. This is in line with the results of previous studies,^20,44,45^ which indicated a higher burden when no support was received from the social network, probably because no back-up is available from family or friends.

All relationship factors included in our study were associated with perceiving burden. This study, like the previous work by some,^26,46^ showed that if more tasks were carried out by informal caregivers, especially personal and medical care tasks in both samples and emotional support in the senior sample, a moderate association was found with perceiving high burden. It was shown that people providing personal/medical care can feel uncomfortable or unable to provide this type of care,^47^ and it was indicated that senior people who provide emotional support might not feel highly valued and respected by their care recipient, which increases their burden.^48^ It was found that more hours of care and a longer duration of the caregiving relation were associated with a high burden.^27,48^ In the current study, only the number of caring hours had a significant effect, which is in agreement with the findings in the study by Kenny, King, and Hall.^49^ Although a poor perceived physical health status showed no significant association in our logistic regression analysis, this factor should be included in future assessments, as the presence of health problems may impede caregivers in providing care.^7,15,20^ A higher number of depressive symptoms was strongly associated with perceiving a high burden. Caregivers have to deal with psychosocial strains because of their caregiving situation and people who are less able to deal with this tend to perceive a higher burden.^20^

Favorable coping variables, that is, mastery and self-management, were suggested to mediate the studied relationship by reducing the perceived burden among caregivers. This was confirmed, where being able to manage your situation, as an informal caregiver, was associated with perceiving a low burden. In particular, caregivers with a high sense of mastery notice a positive influence. This indicates that when caregivers feel able to handle their care provision tasks, show a confrontational coping style, and have a good personal balance between their role as informal caregiver and their personal life, they are more likely to deal successfully with challenges associated with the provision of informal care.^19,20,50^

Significant associations were found between the need for support among informal caregivers and perceiving a high burden. Expressed needs are typically a consequence of the intensity of the care provided, but might also reflect the burden perceived.^51^ Although needs for information and advice and relaxing activities are frequently mentioned, this study showed that reporting a need for replacement was associated with perceiving a high burden. The stronger association may imply that the caregiver is not able to handle the caregiving tasks.^52^ Finally, reporting a need for advocacy was strongly associated with perceiving a high burden, probably because informal caregiving is unrecognized and unsupported by society, in particular caregivers need more support from national and local government, the medical sector, and their employers.^53,54^ By focusing on the needs replacement and advocacy, informal caregivers perceiving a high burden can be supported to be able to provide informal care in the future.

The factors studied explained 30.2% of the variance in the burden perceived by the adult informal caregivers and 36.1% of the variance in the burden perceived by senior caregivers, indicating a small to moderate contribution to a high burden.^55^ Even though the model that was used in the regression analysis was not complete, a relatively large percentage of the variance was explained. Variables such as perceived appreciation for the caregiving tasks, uplifts of caregiving, and the possibility to engage in activities distracting from the caregiving tasks were not included in this study. If it were possible to include all these variables as well, the percentage of explained variance could increase even further.

### Strengths and Limitations

The major strength of this study is the large sample size and the inclusion of a large number of potentially important explanatory variables. The study was sufficiently powered to test multiple associations^56^ and provides a comprehensive overview of the current knowledge and relevant concepts regarding the characteristics and needs of informal caregivers.^57^

Besides these strengths, some limitations should be acknowledged. The cross-sectional design made it impossible to draw conclusions about causality, as it is unknown whether the selected characteristics actually preceded the occurrence of burden.^58^ Second, there was a risk of selection bias in view of the voluntary participation, where some characteristics of the participants may have differed from those of nonparticipants. For example, people with a poorer general health status and with a low level of literacy are less likely to participate in studies like this, and participants who do not perceive themselves to be informal caregivers will not be included either.^59^ Third, the data source consisted of self-reports, which is attended by increased risks of incorrect answers due to information bias. Caregivers might not be willing to share the burden they perceive, which may lead to inaccurate or socially desirable answers, and probably an underestimation of the actual burden on caregivers.^60^ Fourth, although most characteristics were measured by a validated scale, some were not, eg, quality of life and perceived burden. These characteristics were measured on a Likert-type scale and eventually dichotomized. This was necessary to have sufficient numbers of participants in each subgroup, but might also lose some details. Fifth, the data used were gathered in 2012, which means that this study might underestimate the current level of burden being perceived and the associations, because the role of informal caregivers in the Dutch society has increased in recent years. Finally, there is the issue of generalizability, as only caregivers from the Dutch province of Limburg were included. The characteristics of informal caregivers can vary between areas. For instance, it is known that the general health status of the Limburg population is poorer and incomes are lower than in other areas of the Netherlands.^61^

### Recommendations for Future Research and Practice

Based on our findings, 2 main recommendations for future research can be offered. First, longitudinal research is warranted to establish the causal directions of the found associations. This also provides better opportunities to develop a model, which can explain the risk factors for perceiving a high burden and also gives a better insight in the consequence of this burden. Furthermore, explorative research is needed to assess the importance of factors such as perceived appreciation for the caregiving tasks, uplifts of caregiving, and the possibility to engage in activities distracting from the caregiving tasks, as these were not included in the present study. If the importance of these characteristics can be confirmed, they may serve as screening criteria for selecting caregivers who might be at risk of overburdening. In consultation with caregivers who perceive a low or high burden, activities could then be developed to provide support tailored to their needs.

For now, it seems that it is especially those informal caregivers spending more than 16 hours a week providing care who perceive the highest burden. In practice, possibilities should be developed to share the care tasks by involving other family members, while neighbors and friends might also take over some tasks, which might relieve the burden perceived by informal caregivers. Finally, the options should be searched to have a better cooperation with and support from the concerning municipalities and formal care providers, who might also be able to relieve or reduce the burden of informal caregivers.

## Conclusion

This cross-sectional study explored the population characteristics and needs of informal caregivers reporting a low or high burden. In less intensive stages of caregiving, informal care is often perceived to be a source of positive influence, while as the intensity of the care increases, more caregivers report to perceive high burdens. Different risk factors for perceiving a high burden could be identified: female gender, being aged 40 to 54 years, being widowed, providing emotional support, providing many hours of care, low sense of mastery, presence of depressive symptoms, and severe loneliness. Furthermore, caregivers reporting a need for replacement and advocacy may be the ones most at risk for perceiving a high burden. Although longitudinal research is warranted to establish the causal directions of these associations, focusing on these characteristics and needs is useful to relieve the perceived burden of informal caregivers, where a better cooperation with and support from the concerning partners in the municipalities is a necessity.
